# Factors shaping the gut microbiome of five species of lizards from different habitats

**DOI:** 10.7717/peerj.15146

**Published:** 2023-05-09

**Authors:** Diana S. Vasconcelos, D. James Harris, Isabel Damas-Moreira, Ana Pereira, Raquel Xavier

**Affiliations:** 1 CIBIO - Centro de Investigação em Biodiversidade e Recursos Genéticos, InBIO Laboratório Associado, Campus de Vairão da Universidade do Porto, Vairão, Portugal; 2 Departamento de Biologia, Faculdade de Ciências da Universidade do Porto, Porto, Portugal; 3 BIOPOLIS Program in Genomics, Biodiversity and Land Planning, CIBIO - Campus de Vairão, Vairão, Portugal; 4 Department of Behavioural Ecology, Bielefeld University, Bielefeld, Germany

**Keywords:** Metabarcoding, *Podarcis*, *Teira*, Gut microbiota, Bacterial transmission, Sympatry

## Abstract

**Background:**

Host-gut microbiota interactions are complex and can have a profound impact on the ecology and evolution of both counterparts. Several host traits such as systematics, diet and social behavior, and external factors such as prey availability and local environment are known to influence the composition and diversity of the gut microbiota.

**Methods:**

In this study, we investigate the influence of systematics, sex, host size, and locality/habitat on gut microbiota diversity in five lizard species from two different sites in Portugal: *Podarcis bocagei* and *Podarcis lusitanicus*, living in syntopy in a rural area in northern Portugal (Moledo); the invasive *Podarcis siculus* and the native *Podarcis virescens*, living in sympatry in an urbanized environment (Lisbon); and the invasive *Teira dugesii* also living in an urban area (Lisbon). We also infer the potential microbial transmission occurring between species living in sympatry and syntopy. To achieve these goals, we use a metabarcoding approach to characterize the bacterial communities from the cloaca of lizards, sequencing the V4 region of the 16S rRNA.

**Results:**

Habitat/locality was an important factor explaining differences in gut bacterial composition and structure, with species from urbanized environments having higher bacterial diversity. Host systematics (*i.e.*, species) influenced gut bacterial community structure only in lizards from the urbanized environment. We also detected a significant positive correlation between lizard size and gut bacterial alpha-diversity in the invasive species *P. siculus*, which could be due to its higher exploratory behavior. Moreover, estimates of bacterial transmission indicate that *P. siculus* may have acquired a high proportion of local microbiota after its introduction. These findings confirm that a diverse array of host and environmental factors can influence lizards’ gut microbiota.

## Introduction

A myriad of microorganisms can be found living in the gastrointestinal tract of all animals. Studies on gut microbiome dynamics have shown that gut microbial assemblages are composed by host-adapted ‘core’ microbial taxa as well as a mixture of transient microbes (Shapira, 2016). These microbial commensals are known to have a significant impact on host biology, influencing a variety of processes that affect host fitness (Shapira, 2016; Cryan & Dinan, 2012; Thaiss et al., 2016), with the hologenome theory of evolution being recently proposed, where selection acts upon host genome and its associated microbiome as a single evolutionary unit (Zilber-Rosenberg & Rosenberg, 2008). The gut microbiome is known to be involved in disease resistance, besides being important for xenobiotics metabolism, nutrient uptake and energy acquisition (*e.g.*, Vavre & Kremer, 2014; Rowland et al., 2018). Moreover, gut microbiota may also contribute towards host adaptation to environment changes by enabling a response to new challenges, such as exploitation of novel food sources (Delsuc et al., 2014; Hammer & Bowers, 2015). Ultimately, the gut microbiome may have a major impact on host development, behaviour and fitness, with cascading effects to the dynamics of ecosystems (Thaiss et al., 2016; Cusick, Wellman & Demas, 2021; Jia et al., 2021; Chiang et al., 2022). In turn, gut microbial communities may also be modulated by various host traits, such as host evolutionary history, sex and size (Youngblut et al., 2019; Song et al., 2020; Bunker, Arnold & Weiss, 2022). External environment, such as habitat or prey availability also affect the gut microbiome of vertebrate species (*e.g.*, Muegge et al., 2011; Xavier et al., 2019; Montoya-Ciriaco et al., 2020; Jin et al., 2021). In addition, social interactions between hosts can also influence the gut microbiome in many animal species, although these mechanisms remain less studied (see review by Archie & Tung, 2015).

Gut microbiome studies have been performed in many mammals (*e.g.*, Thaiss et al., 2016), birds (*e.g.*, Hird et al., 2015), fishes (*e.g.*, (Xavier et al., 2020)) and amphibians (*e.g.*, Bletz et al., 2016). Comparatively, only a few have been performed in reptiles, and only a handful of these addressed lizards. Nevertheless, studies showed that maternal transmission of gut microbiota to offspring can occur in squamate reptiles (Kohl et al., 2017). Additionally, microbiota can be acquired by reptiles through horizontal transmission from the environment or through interaction with other organisms (*e.g.*, predatory encounters, Kohl et al., 2017). Host systematics, and ecology were also seen to be important drivers of gut microbiota diversity in reptiles (*e.g.*, Hong et al., 2011; Smith, Colston & Siler, 2021). For example, feeding habits influence the gut microbiota of the Chinese crocodile lizard, *Shinisaurus crocodilurus* Ahl 1930, with potential effects on host health due to the influence of diet on the abundances of pathogenic or opportunistic gut bacteria (Jiang et al., 2017). Trophic niche was also reported to be an important determinant of the gut bacterial community structure, with differences found between herbivorous and omnivorous lizard species and specific bacterial taxa being linked to adaptation to herbivory (Kohl et al., 2017). The diet and habitat of the Australian water dragon, *Intellagama lesueurii* (Gray, 1831), also had an effect on its gut microbiome, with lizards living in urban areas presenting higher bacterial diversity than populations living in natural habitats (Littleford-Colquhoun et al., 2019). Other habitat characteristics were also seen to influence lizard microbiome. For example, altitude was an important factor explaining community composition and structure of the gut microbiota of the lizard *Phrynocephalus vlangalii* (Zhang et al., 2018) and temperature changes also altered the composition of the common lizard (*Zootoca vivipara*) gut microbiome, with warming temperatures resulting in a decrease in diversity and an increase in pathogenic bacteria (Bestion et al., 2017).

Here, we analyze and compare the diversity, composition and structure of gut bacterial communities of five related lacertid species captured in Portugal. Individuals of *Podarcis bocagei* (Lopez-Seoane, 1885) and *P. lusitanicus* Geniez et al., 2014 were sampled in Moledo (North of Portugal) where they live in syntopy. Invasive *P. siculus* (Rafinesque-Schmaltz, 1810) and native *P. virescens* Geniez et al., 2014 were sampled from Parque das Nações (Lisbon) where they live in sympatry. Finally, a population of the introduced *Teira dugesii* (Milne-Edwards, 1829) was sampled in the Alcantara Docks in Lisbon. All five species exhibit sexual dimorphism, with males usually being larger than females. They are mostly insectivorous (Geniez et al., 2014; Carretero, Galán & Salvador, 2015), although *P. siculus* and *T. dugesii* may also occasionally consume some fruits or flowers (Mačát, Veselý & Jablonski, 2015). *Podarcis* species are considered model organisms to study ecotoxicology, immune/histochemical reactions, among other processes (*e.g.*, Bicho et al., 2013; Luís et al., 2019); however, microbiome studies are still largely lacking, with only three studies available to date. Two studies investigated two species endemic to the Balearic Islands (Spain), *P. lilfordi* (Günther, 1874) and *P. pityusensis* (Bosca, 1883), with results indicating that islet, time since separation from mainland, and seasonality are significant factors contributing to their gut microbiome (Baldo et al., 2018; Alemany et al., 2022). Another recent study compared the gut microbiota of two Italian populations of *P. siculus* (mainland *vs* island) demonstrating that there were considerable differences between the two (Buglione et al., 2022).

Our main objective was to determine whether locality, which also corresponded to two different habitats (rural *vs* urbanized), and host factors such as species, size, and sex, modulate the gut bacterial diversity of these five lizards. To achieve this, we used cloacal swabs to obtain a proxy for gut bacterial communities, which were characterized by sequencing the V4 region of the 16S rRNA gene. Swabs were preferred to fecal samples as these more accurately reflect microbial communities residing in lower gut and cloacal tissues (Bunker, Martin & Weiss, 2022).

## Methods

### Sample collection

**Figure 1:**
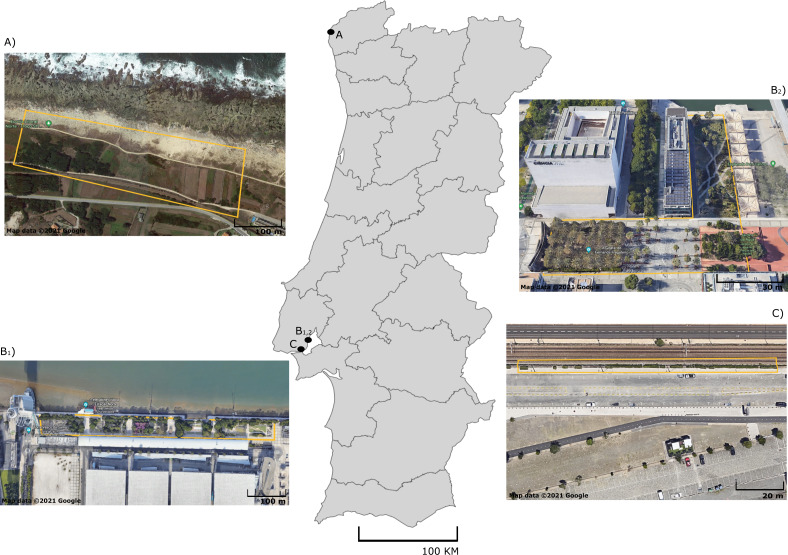
Sampling sites of five lizard species from Portugal. Map of Portugal with aerial photographs of sampling sites for (A) *P. bocagei* and *P. lusitanicus* (Moledo), B_1_) P. virescens, B_2_) *P. siculus* (Parque das Nações) and (C) *T. dugesii* (Alcantara docks). Specific collection areas are delimited by yellow lines. Map data ©2021 Google.

A total of 100 adult lizards from five different species were sampled in September 2020: *Podarcis bocagei* (males = 22; females = 9), *P. lusitanicus* (males = 6; females = 2), *P. siculus* (males = 13; females = 6), *P. virescens* (males = 16; females = 6), and *Teira dugesii* (males = 7; females = 13) (Table S1). These lacertid species are small-sized, with captured individuals measuring on average 6.96 cm snout-vent length (SVL) in *P. siculus*, 5.55 cm in *P. virescens*, 5.03 cm in *P. bocagei*, 4.65 cm in *P. lusitanicus*, and 6.06 cm in the introduced species *T. dugesii* (Table S1). *Podarcis bocagei* and *P. lusitanicus* were collected on 28–29th of September 2020 from a semi-natural habitat in Moledo, northern Portugal (Fig. 1A) (41°50′19.2″N 8°52′24.5″W), where they live in syntopy (*i.e.,* occurrence of two species in the same habitat at the same time). This location has limited human disturbance, just a few meters from the beach and has extensive vegetation with natural and artificial shelters (*e.g.*, walls of agricultural properties) that can be used by lizards. Ecological adaptation is considered a major factor favoring the isolation between these two species; *P. lusitanicus* lives more on rocks, while *P. bocagei* is ground-dwelling (Carretero, Galán & Salvador, 2015). The diet of these two species is mainly composed by prey belonging to Hemiptera, Coleoptera, Diptera, Hymenoptera and Araneae, with minimal differences between species or sexes (Kaliontzopoulou et al., 2011). *Podarcis siculus* and *P. virescens* were collected on 15–16th of September 2020 in Lisbon, at Parque das Nações (Figs. 1B_1_, B_2_) (38°45′43.6″N 9°05′40.2″W; 38°46′10.6″N 9°05′31.5″W), where both live in sympatry (sharing habitat type). This is a highly urbanized area near the Tejo River, characterized by large residential and commercial areas, with considerable daily human disturbance. While *P. virescens* is native to this location, *P. siculus* is native to the Italian Peninsula, east Adriatic coast and many nearby islands, being invasive elsewhere, including in Lisbon where it was likely introduced about 25 years ago (Gonzálezdela Vega, García-Pulido & González-García, 2001; Silva-Rocha, Salvi & Carretero, 2012). Its plasticity in spatial use of habitat, morphology, behaviour, and diet explains its successful colonization of multiple locations outside its native range (Vervust et al., 2010; Carretero & Silva-Rocha, 2015; Damas-Moreira et al., 2019; Damas-Moreira et al., 2020). This invasive species can present a more versatile diet, as it can also consume fruits and nectar (Mačát, Veselý & Jablonski, 2015; Vervust et al., 2010), while *P. virescens* is known to be insectivorous and to feed mainly on individuals of the class Arachnida and the orders Hymenoptera, Hemiptera, Coleoptera, and Diptera (Juan, 1997). Finally, we collected *T. dugesii* in a nearby area in Lisbon on the 16th of September 2020, in the Alcantara docks, close to the city port area (Fig. 1C) (38°42′12.0″N 9°09′55.9″W). Similar to the other *Podarcis* spp. captured in Lisbon, *T. dugesii* occupies an anthropogenic area, although less busy, close to railway tracks with limited vegetation cover (Fig. 1C). This species was likely introduced by accident *via* transport ships from Madeira Island three decades ago, in 1992 (Sá-Sousa, 1995). *Teira dugesii* feeds preferentially on insects but also on small fruits (Sadek, 1981). The average monthly temperature and precipitation in September 2020 was 20–18 °C and 50–100 mm, respectively, in Moledo and 20–22 °C and 25–100 mm in Lisbon (Portuguese Institute of Sea and Atmosphere, 2020).

Experimental protocols and research were approved by the Portuguese Institute for Conservation of Nature and Forests (ICNF) (License 703/2021/CAPT). No animal experimentation was performed in this study, and all samples were collected non-invasively using sterile cotton swabs (Dryswabs™, Medical Wire and Equipment, Wiltshire, UK). All individuals were captured using nooses. Lizards were carefully immobilized, avoiding any human contact with the cloaca. We quickly inserted a sterile cotton swab into the entrance of the cloaca to obtain individual microbial samples. The tips of the swabs were cut into individual tubes and stored immediately in ice boxes in the field, and frozen at −20 °C within a maximum of 5 h after sampling. After finishing the field work, swabs were taken to the laboratory for storage at −80 °C until processing. After microbial sampling, each lizard was sexed, and the snout-vent length was measured (SVL; from head to cloaca) using a digital caliper (± 0.01 mm error). No animals died or were euthanized during sampling and all animals were released unharmed at the place of collection.

### DNA extraction and sequencing

In the laboratory, DNA was extracted from the swabs using the DNeasy^®^ PowerSoil^®^ Kit (Qiagen, Hilden, Germany) according to the manufacturer’s instructions. DNA concentration and quality were measured with the Epoch™ Microplate Spectrophotometer (BioTek Instruments, Inc.; Winooski, VT, USA). DNA was shipped in dry ice to the Centre for Microbial Systems at the University of Michigan Medical School (USA) where the V4 region of the 16S rRNA gene (∼250 bp) of the bacterial communities was amplified for each sample, along with the extraction blanks and PCR controls using the primers 515F (5′-GTGCCAGCMGCCGCGGTAA-3′) and 806R (5′-GGACTACHVGGGTWTCTAAT −3′) and following the protocol of Kozich et al. (2013). The V4 region of this gene is widely used to characterize bacterial communities in various taxa, including reptiles (*e.g.*, Colston & Jackson, 2016; Chiarello et al., 2018). Amplicons were sequenced in a single Illumina MiSeq run using a MiSeq Reagent Kit V2 500 cycles. Raw sequence reads were deposited into NCBI’s Short Read Archive under project PRJNA895230.

### Sequence and statistical analysis

All analyses were performed using the R Software v.4.1.1 (R Core Team, 2020). Raw FASTQ files were denoised using the DADA2 pipeline (Callahan et al., 2016). After an assessment of read quality plots, the parameters for trimming and filtering were set as: trimLeft = 20, truncLen = c(220, 200), maxN = 0, maxEE = c(2, 2), truncQ = 2. The SILVA 138 database (Pruesse et al., 2007; Quast et al., 2013) was chosen for taxonomic assignment. After quality control and taxonomic assignment, sequences identified as Archaea, Eukaryota, Mitochondria, Chloroplast, as well as sequences unassigned to Bacteria, were removed from the dataset. ASVs present in control samples were also removed from downstream analysis. An amplicon sequence variant (ASV) frequency table was constructed using the R package *phyloseq* (McMurdie & Holmes, 2013). Normalized read counts were obtained using the negative binomial distribution implemented in DESeq2 (Love, Huber & Anders, 2014; McMurdie & Holmes, 2014). We removed ASVs that concomitantly met the two following criteria: had a count of less than 0.001% of the total number of reads (3,586,752 [total number of reads] × 0.001% = 36) and that were also present in a single sample (Table S2). The composition and abundance of taxa in the mock community were similar to those described by the manufacturer.

Bacterial diversity (alpha-diversity, calculated intra-sample) and structure (beta-diversity, calculated as the dissimilarity or distance between pairs of samples) were estimated using the *phyloseq* and the *picante* packages (McMurdie & Holmes, 2013; Kembel et al., 2010) (see R script provided as supplementary materials). Alpha-diversity was estimated using the number of observed ASVs, the Shannon index, and Faith’s Phylogenetic Diversity (PD). Beta-diversity was measured using the Bray–Curtis index and the Unifrac phylogenetic weighted and unweighted distances. Principal coordinate analysis (PCoA) were used to visually assess dissimilarity among groups.

Statistical differences in alpha-diversity between localities were assessed using species as a random factor using a linear mixed effects model (lmer(alpha-diversity ∼ locality + (1—species)) with the package *lme4* (Bates et al., 2014). Given the significant effect of locality on alpha-diversity (see results section), differences in alpha-diversity among species and between sexes were further assessed using another linear mixed effects model with locality as a random factor (lmer(alpha-diversity ∼ species + species/sex + (1—locality)). The effects of locality and species on microbial beta-diversity were assessed using a permutational analysis of variance (PERMANOVA) with 9,999 permutations, with the *adonis2* function of the R *vegan* package (Oksanen et al., 2013), using the formula (adonis2(beta-diversity ∼ locality + species)). Since both locality and species significantly affected beta-diversity, the pairwise effects of species and sex were tested for each locality separately using the *pairwise.adonis2* function (Arbizu, 2020) using the model (pairwise.adonis2(beta-diversity ∼ species + species/sex)). *P*-values for multiple comparisons were adjusted with the Bonferroni correction. Differences in the proportions of the most abundant taxa at the phyla and genera levels (represented by ≥ 3% on average of all sequences) were assessed between species and sex for each locality separately using a linear model (lm(bacterial taxa ∼ species + species/sex)). Correlations between individual size and bacterial alpha-diversity were also tested using the Pearson correlation test for each species, using the *ggpubr* package (Kassambara & Kassambara, 2020) (see Table S1).

To further understand the levels of similarity between sympatric and syntopic species, bi-directional bacterial transmission between each pair of species from Moledo and Parque das Nações was estimated using the FEAST software (Shenhav et al., 2019), by testing the contribution of each species (source) to the microbial diversity to its sympatric congener (sink). To this end, the non-normalized ASV frequency table was used and, due to differences in the number of samples between *P. bocagei* and *P. lusitanicus,* only a fraction of the individuals of *P. bocagei* were included (the ones with the most similar sex and SVL ratios to the *P. lusitanicus* samples as possible, with included *P. bocagei* individuals having a mean SVL of 4.6 cm *vs P. lusitanicus* with a mean SVL of 4.7 cm), following the FEAST developers’ recommendations to avoid overestimation of transmission.

## Results

After filtering, the final ASV table encompassed 3,923 unique ASVs, included in a total of 39 bacteria phyla. The most abundant phyla among the species studied were Firmicutes, Bacteroidota, Actinobacteroidota, Proteobacteroidota and Campylobacterota.

**Figure 2:**
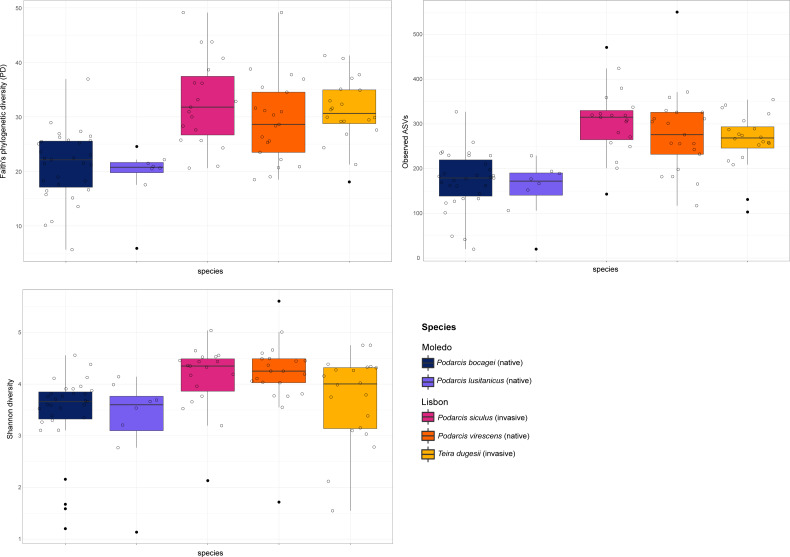
Boxplots of alpha-diversity of the studied lizards’ gut microbiome. Boxplots of the alpha-diversity indices (Faith’s phylogenetic diversity, Shannon diversity and the number of observed ASVs) for the gut microbiome of the studied lizards. Lines within each boxplot indicate the median and whiskers display the range. Open dots represent data points and black dots represent outliers.

**Figure 3:**
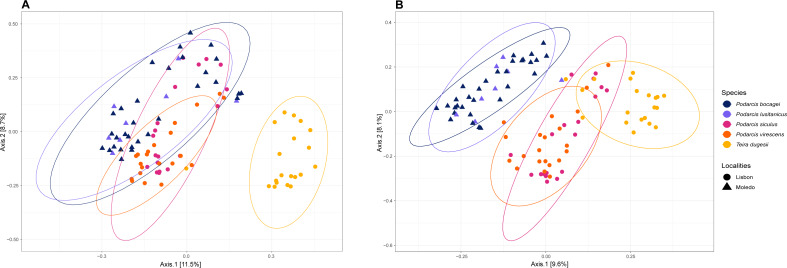
Principal coordinate analysis (PCoA) plots of the gut bacterial beta-diversity of the studied lizards. PCoA plots representing Bray–Curtis (A) and Unweighted Unifrac (B) distances, grouped by lizard species with a 95% confidence interval ellipse.

Gut bacterial diversity, measured through alpha-diversity indices, was significantly different between localities considering the number of observed ASVs and PD indices (observed ASVs: *F*-statistics = 39.74, DF = 1, *p* = 0.02; PD: *F*-statistics = 51.38, DF = 1, *p* = 0.02), with species from Lisbon showing consistently higher alpha-diversity indices than the ones from Moledo (Fig. 2). No differences between localities were found with the Shannon index (*F*-statistics = 8.33, DF = 1, *p* = 0.07). Moreover, neither species or sex had a significant effect on microbial alpha-diversity (*F* > 1.03, DF = 4, *p* > 0.11 and *F* > 0.57, DF = 5, *p* > 0.16, respectively), although *P. siculus* had higher diversity than the native *P. virescens*. Microbial structure, measured through beta-diversity indices, was significantly different between localities (Bray–Curtis: *R*^2^ = 0.07, DF = 1, *p* = 0.0001; Weighted Unifrac: *R*^2^ = 0.03, DF = 1, *p* = 0.04; Unweighted Unifrac: *R*^2^ = 0.08, DF = 1, *p* = 0.0001) and species (Bray-Curtis: *R*^2^ = 0.12, DF = 3, *p* = 0.0001; Weighted Unifrac: *R*^2^ = 0.08, DF = 3, *p* = 0.03; Unweighted Unifrac: *R*^2^ = 0.11, DF = 3, *p* = 0.0001) (Fig. 3). In general, pairwise differences in beta-diversity between species were found in species collected from Lisbon (Table 1), while no differences were found between sexes. In samples collected in Moledo, no differences were found in beta-diversity between species or sexes.

**Table 1:** Results from the pairwise PERMANOVA testing the effect of species and sex in gut microbial beta-diversity. Results presenting R^2^ and respective adjusted *p*-values. Significant results are depicted in bold.

			**Unifrac phylogenetic weighted**	**Unifrac phylogenetic unweighted**	**Bray–Curtis**
**Lisbon**	*Podarcis siculus* vs. *Podarcis virescens*	species	***R***^**2**^ = **0.10**; ***p*** = **0.03**	***R***^**2**^ = **0.18**; ***p*** = **0.0003**	***R***^**2**^ = **0.12**; ***p*** = **0.003**
		sex	*R*^2^ = 0.01; *p* = 0.99	***R***^**2**^ = **0.11**; ***p*** = **0.02**	*R*^2^ = 0.06; *p* = 0.24
	*Podarcis siculus* vs. *Teira dugesii*	species	*R*^2^ = 0.03; *p* = 0.37	***R***^**2**^ = **0.44**; ***p*** = **0.0001**	***R***^**2**^ = **0.49**; ***p*** = **0.0001**
		sex	R^2^ = 0.01; *p* = 0.98	***R***^**2**^ = **0.07**; ***p*** = **0.04**	*R*^2^ = 0.04; *p* = 0.17
	*Podarcis virescens* vs. *Teira dugesii*	species	***R***^**2**^ = **0.10**; ***p*** = **0.01**	***R***^**2**^ = **0.45**; ***p*** = **0.0001**	***R***^**2**^ = **0.52**; ***p*** = **0.0001**
		sex	*R*^2^ = 0.02; *p* = 0.90	*R*^2^ = 0.06; *p* = 0.06	*R*^2^ = 0.02; *p* = 0.44
**Moledo**	*Podarcis bocagei* vs. *Podarcis lusitanicus*	species	*R*^2^ = 0.02; *p* = 0.41	*R*^2^ = 0.02; *p* = 0.92	*R*^2^ = 0.02; *p* = 0.75
		sex	*R*^2^ = 0.05; *p* = 0.41	*R*^2^ = 0.06; *p* = 0.26	*R*^2^ = 0.05; *p* = 0.73

**Figure 4:**
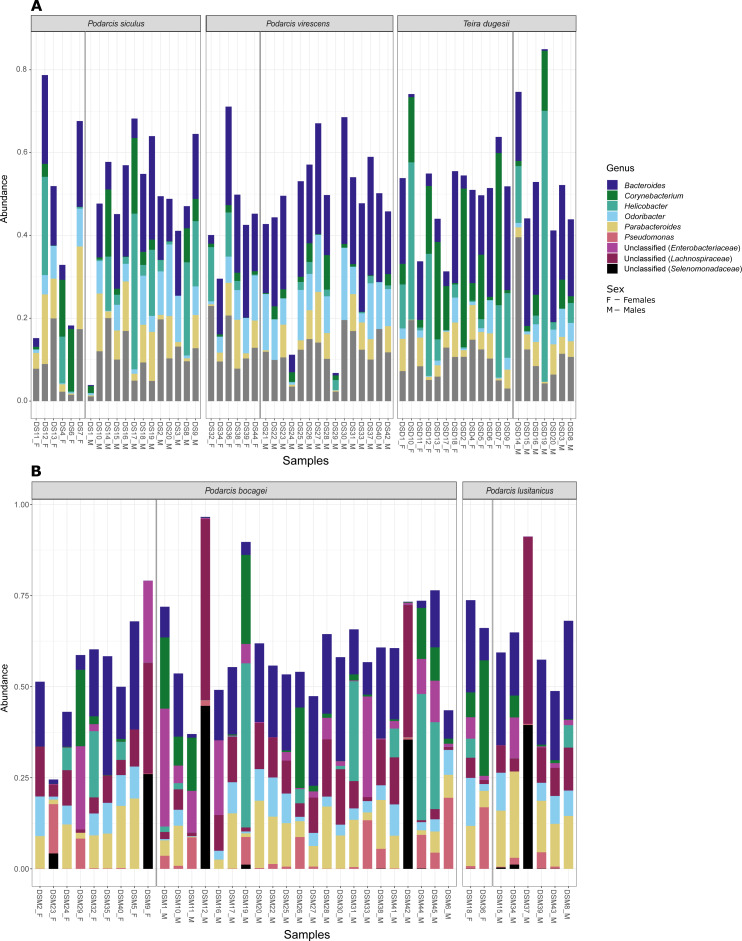
Most abundant bacterial genera from the gut of the five studied lizard species. Relative abundance of the most abundant bacterial genera (≥ 3% on average of all sequences) in the gut microbiome of the studied lizard species from Lisbon (A) and from Moledo (B).

**Figure 5:**
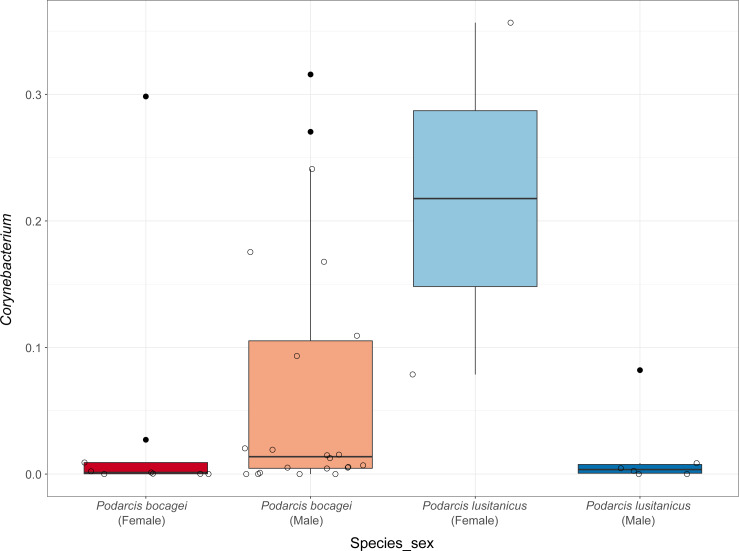
Relative abundance of *Corynebacterium* in the gut microbiota of species from Moledo between sexes. Differences in the proportion of the most abundant genus *Corynebacterium* between males and females of *P. bocagei* and *P. lusitanicus*. Lines within each boxplot indicate the median and whiskers display the range. Open dots represent datapoints and black dots represent outliers.

Although no differences were found in the proportion of the most abundant phyla between species or sexes, some differences were observed when considering the most abundant genera (Fig. 4). In the case of species in Moledo, sex influenced the proportion of the genus *Corynebacterium* (*F*-statistics = 4.46, DF = 3, *p* = 0.02) (Table 2, Fig. 5). Moreover, differences between *P. siculus* and *P. virescens* were also found for genus *Corynebacterium* (*F*-statistics = 6.66, DF = 2, *p* = 0.003) and for *Odoribacter* (*F*-statistics = 10.10, DF = 2, *p* = 0.0002) (Table 2).

**Figure 6:**
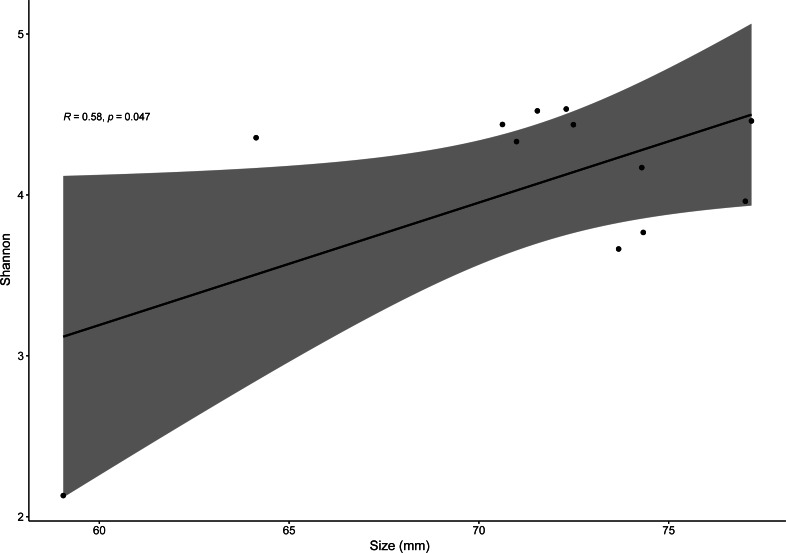
Linear regression for effects of size on bacterial diversity. Linear regression plot between size (SVL) and gut bacterial alpha-diversity (Shannon index) for *P. siculus*. The colored area represents a 95% confidence limit.

Pearson correlation test only showed significantly positive correlations between SVL and bacterial alpha-diversity (for Shannon index, *R* = 0.58, *p* = 0.04) for males of the invasive species *P. siculus* (Fig. 6). For all other species, *R* values varied from −0.082 to 0.68 with *p* > 0.05. It is important to note that the significant positive correlation of males of *P. siculus* was reversed when the smallest sized individual (59 mm) was removed from the dataset (R = −0.62, *p* = 0.04).

Results from FEAST software indicated that the level of bacterial transmission between sympatric species in both populations (Parque das Nações and Moledo) was high. Nevertheless, while bacterial transmission was balanced in both directions between the syntopic *P. lusitanicus* and *P. bocagei* (estimated transmission from *P. bocagei* towards *P. lusitanicus* was ∼71% on average, and from *P. lusitanicus* towards *P. bocagei* was ∼69% on average), between the two sympatric species in Lisbon there was a more biased transmission, with *P. virescens* seemingly having a higher contribution towards *P. siculus* gut microbiota (transmission estimates from *P. virescens* towards *P. siculus* was ∼72% on average, and from *P. siculus* towards *P. virescens* it was ∼55% on average).

## Discussion

In this study, we characterized the gut bacterial microbiota of five lizard species from Portugal (the native *Podarcis virescens*, *P. bocagei* and *P. lusitanicus,* and the introduced *P. siculus* and *Teira dugesii*) using a metabarcoding approach. The most abundant phyla found in the lizard species studied herein were Firmicutes, Bacteroidota, Actinobacteroidota, Proteobacteroidota, and Campylobacterota, with these results being in agreement with what has been found in other studies in lizards using cloaca swabs (Jiang et al., 2017; Bunker, Martin & Weiss, 2022). Likewise, some of the most abundant genera found in the present study (Table 2) have also been shown to be highly abundant in the gut of other lizard species (*e.g.*, *Bacteroides*, *Odoribacter*, and *Parabacteroides*, Zhang et al., 2018; Montoya-Ciriaco et al., 2020). It is noteworthy that the gut microbiota of individuals of *P. siculus* sequenced herein and those from Italy by Buglione et al. (2022) share the most abundant bacterial phyla but differ at the level of the most represented bacterial genera.

**Table 2:** Results from the linear models testing the effect of species and sex in the proportion of the most abundant genera for each locality. Family of genera that remained unclassified is presented between brackets. Significant results are depicted in bold.

	**Lisbon**		**Moledo**	
	Species (DF = 2)	Sex (DF = 3)	Species (DF = 1)	Sex (DF = 2)
***Odoribacter***	***F*** = **10.10**; ***p*** = **0.0002**	*F* = 1.16; *p* = 0.33	*F* = 0.72; *p* = 0.40	*F* = 0.81; *p* = 0.45
***Corynebacterium***	***F*** = **6.66**; ***p*** = **0.003**	*F* = 2.09; *p* = 0.11	*F* = 0.13; *p* = 0.72	***F*** = **4.46**; ***p*** = **0.02**
*Helicobacter*	*F* = 2.96; *p* = 0.06	*F* = 0.32; *p* = 0.82	*F* = 1.01; *p* = 0.32	*F* = 0.35; *p* = 0.71
*Parabacteroides*	*F* = 2.11; *p* = 0.13	*F* = 0.53; *p* = 0.67	*F* = 2.53; *p* = 0.12	*F* = 0.68; *p* = 0.51
*Bacteroides*	*F* = 2.43; *p* = 0.10	*F* = 1.11; *p* = 0.35	*F* = 1.33; *p* = 0.26	*F* = 0.03; *p* = 0.97
*Pseudomonas*	NA	NA	*F* = 0.04; *p* = 0.85	*F* = 1.88; *p* = 0.17
Unclassified (*Selenomonadaceae*)	NA	NA	*F* = 0.11; *p* = 0.74	*F* = 0,26; *p* = 0.76
Unclassified (*Lachnospiraceae*)	*F* = 1.42; *p* = 0.25	*F* = 0.18; *p* = 0.91	*F* = 0.18; *p* = 0.68	*F* = 0.76; *p* = 0.48
Unclassified (*Enterobacteriaceae*)	*F* = 0.27; *p* = 0.77	*F* = 0.66; *p* = 0.58	*F* = 0.93; *p* = 0.34	*F* = 0.06; *p* = 0.94

Our results showed that locality was a major predictor of microbial diversity and structure. Plausibly, differences in habitat may lead to differences in the composition and diversity of the gut microbiome of animals (*e.g.*, Amato et al., 2013; Xavier et al., 2021). The two habitats in which the lizards from this study were captured are very different, with lizards from Lisbon living in a highly urbanized and artificial habitat, with greater environmental disturbance, compared to lizards from Moledo, which live in a semi-natural habitat. Specifically, we detected a consistently higher microbial diversity in the species from Lisbon which could possibly be explained by the higher variety of diet items consumed. *Podarcis siculus* diet is viewed as extremely opportunistic, and can include fruits, other lizards, small carrion and even human food waste (*e.g.*, cheese and pasta) (Mo & Mo, 2021; Mačát, Veselý & Jablonski, 2015; Capula & Aloise, 2011). Additionally, we cannot discard a potential influence of climatic variability between the two localities.

Although the species we sampled in urban areas can also reside in rural habitats, and vice-versa, our sampling design did not allow comparisons of gut bacterial communities between conspecifics residing in these two habitats. Nevertheless, urbanization is frequently seen to restructure the gut microbiome of animals (*e.g.*, Stothart, Palme & Newman, 2019), with increases in microbiome alpha-diversity reported for some reptiles (avian and non-avian) and mammals (Dillard et al., 2022). For example, higher gut bacterial diversities were previously reported in a study from populations of the Australian water dragon residing in urban environments when compared to those inhabiting natural environments, presumably driven by differences in the diet (Littleford-Colquhoun et al., 2019). Additionally, authors hypothesized that environmental microbiota, which may be horizontally transferred to lizards, could also be more diverse in urban habitats than in semi-natural ones (Littleford-Colquhoun et al., 2019). A similar pattern was observed in urban crested anole lizards, white-crowned sparrows, as well as in coyotes (Dillard et al., 2022; Phillips, Berlow & Derryberry, 2018; Sugden et al., 2020). Interestingly, Dillard et al. (2022) found increased similarities between the gut microbiota of these three animals and human populations in urbanized habitats. Different hypotheses have been put forward to explain this trend, including that it could be caused by increased heterogeneity of urban land cover (Phillips, Berlow & Derryberry, 2018), higher consumption of human food waste (Sugden et al., 2020) and acquisition of human microbiota in urban habitats (Dillard et al., 2022). We hypothesize that the higher microbiome diversity in lizards from the urban environment could also be related with the aforementioned factors, but further studies including conspecific lizards from urban and natural habitats are needed to determine the generality of this pattern.

Gut microbial diversity (alpha-diversity) and bacterial community structure (beta-diversity) did not differ between the two syntopic species, *P. bocagei* and *P. lusitanicus*, sampled at Moledo. Additionally, our analysis of potential bacterial transmission between these two syntopic lizards indicates a high and balanced bi-directional transmission of bacteria between the two species (ca. 70%), indicating a high similarity between their gut microbiota (Shenhav et al., 2019). This is not surprising as the two species have high dietary overlap and similarity in their habitat occupancy. Moreover, it is likely they consume the same or very similar prey items (Kaliontzopoulou et al., 2011), and also encounter each other frequently. On the contrary, although there were no differences in alpha-diversity between species sampled in Lisbon, species-specific gut bacterial community structure (beta-diversity) were found, indicating ecological differences between these species. Additionally, in comparison with the two species sampled in Moledo, lower and unbalanced estimates for bacterial transmission were uncovered between the two sympatric species, *P. siculus* and *P. virescens*. The invasive *P. siculus* was estimated to receive a higher proportion of bacteria from the native *P. virescens* than vice versa (ca. 72% *vs* 55% on average). These differences could be related to an increased habitat occupancy and successful adaptation to the environment by the invasive species, which facilitated the acquisition of a higher quantity of local microbiota upon its arrival. These results could also be reflecting an increased ability to exploit a variety of food resources, or most likely a combination of both. Although the populations of *P. siculus* and *P. virescens* are found living in sympatry, occupying roughly the same area, they are rarely in syntopy, although sightings of these two species within a few meters of each other have been reported (Ribeiro & Sá-Sousa, 2018). The proportion of some of the most abundant bacterial genera found in our study also differed between *P. virescens* and *P. siculus,* but not between *P. lusitanicus* and *P. bocagei*. The influence of host taxonomy in gut microbiota, which is a proxy not only for host genetics but also its general ecology, has been reported in many animals (Moeller et al., 2013; Moeller et al., 2014), including reptiles (Smith, Colston & Siler, 2021).

Overall, host sex had no effect on microbiome diversity and structure, although the proportion of the genus *Corynebacterium* significantly differed between sexes in species collected from Moledo (*P. bocagei* and *P. lusitanicus*). An increased abundance of *Corynebacterium* may have a potential negative impact on the host, as found in a study on birds where this genus was hypothesised to decrease the reproductive performance of females (Leclaire et al., 2022). Here, females of *P. lusitanicus* (*n* = 2) had a higher abundance of *Corynebacterium* compared to males (*n* = 6) (Fig. 5). However, the number of females sampled in the present study was low, so further studies are warranted to confirm this finding and understand whether these bacteria negatively affect reproductive output of this lizard. Another interesting result was that size of males of *P. siculus* was positively related with bacterial diversity. This lizard can reach larger sizes than the other studied species (Carretero & Silva-Rocha, 2015; Damas-Moreira et al., 2019). Links between lizard size and microbiome diversity have been previously established and explained by increased gut space and longer transit time permitting microbes to have more opportunities of colonisation (Reese & Dunn, 2018). Furthermore, *P. siculus* can also be bolder and more aggressive than native *Podarcis* species (Downes & Bauwens, 2002), and also more exploratory and better at exploiting food resources when compared to the native *P. virescens* at our study location (Damas-Moreira et al., 2019; Damas-Moreira et al., 2020). These behaviors can be associated with the displacement of *P. virescens* from gardens now inhabited by *P. siculus* (Ribeiro & Sá-Sousa, 2018) and can also be leading to a wider ecological and trophic niche, and consequently to the correlation found, as well as to the slightly higher average microbiome diversity observed for *P. siculus*. However, it is important to note that the correlation found between the size of *P. siculus* and gut microbiome diversity is influenced by the smallest individual captured*,* with more studies needed to confirm this tendency.

## Conclusions

The present study contributes to the existing knowledge on the effects of the environment and host factors on the dynamics of the gut microbiome of lizards. Our results showed that habitat was the major driver of differences in microbiome diversity, with lizards from urban environment displaying higher diversity, which probably results from a combination of factors including increased habitat heterogeneity and a potentially more diverse diet. The effect of host taxonomy was more evident in microbiome structure, with differences found between the two sympatric species, *P. siculus* and *P. virescens*, and also between *T. dugesii* and *Podarcis* spp. Although sex had a minor role in determining gut bacterial assemblages of the lizards studied, *Corynebacterium* was more abundant in females of *P. bocagei*, with potentially deleterious effects for reproduction. Additionally, the diversity of gut microbiome of males of *P. siculus* seems to increase with host size. Finally, our results set the stage for future research exploring the influence of diet and urbanization on the microbiome of *Podarcis* and the use of sympatric lizards as models to test the effects of behavior on lizard microbial composition.

##  Supplemental Information

10.7717/peerj.15146/supp-1 Supplemental Information 1 Supplementary Tables

10.7717/peerj.15146/supp-2 Supplemental Information 2 ASV Sequence data

10.7717/peerj.15146/supp-3 Supplemental Information 3 R Script used in microbiome analysis

## Additional Information and Declarations

### Field Study Permissions

The following information was supplied relating to field study approvals (i.e., approving body and any reference numbers): Institute for Conservation of Nature and Forests (ICNF), License 703/2021/CAPT

### DNA Deposition

The following information was supplied regarding the deposition of DNA sequences: The raw sequence reads are available at NCBI’s Short Read Archive: PRJNA895230.

### Data Availability

The following information was supplied regarding data availability: R Script used in microbiome analysis and raw data file with lizard metadata + ASV table.
